# Hydrogen Sulfide Plays an Important Role by Influencing NLRP3 inflammasome

**DOI:** 10.7150/ijbs.47595

**Published:** 2020-08-25

**Authors:** Honggang Wang, Xingzhuo Shi, Mengyuan Qiu, Shuangyu Lv, Hong Zheng, Baohua Niu, Huiyang Liu

**Affiliations:** 1 Institute of Biomedical Informatics, Bioinformatics Center, School of Basic Medical Sciences, Henan University, Kaifeng, Henan, 475000, China; 2 School of Life Science, Henan University, Kaifeng, Henan, 475000, China

**Keywords:** Hydrogen sulfide, NLRP3 inflammasome, renal protection, neuroprotection, diabetes

## Abstract

Inflammasome is a complex composed of several proteins and an important part of the natural immune system. Nucleotide-binding oligomerization domain-like receptor protein 3 (NLRP3) inflammasome is composed of NLRP3, apoptosis associated speck like protein (ASC) and pro-caspase-1. It plays an important role in many diseases. Hydrogen sulfide (H_2_S) is an important signaling molecule that regulates many physiological and pathological processes. Recent studies indicated that H_2_S played anti-inflammatory and pro-inflammatory roles in many diseases through influencing NLRP3 inflammasome, but its mechanism was not fully understood. This article reviewed the progress about the effects of H_2_S on NLRP3 inflammasome and its mechanisms involved in recent years to provide theoretical basis for in-depth study.

## Introduction

A decade ago, inflammasome was described as a large intracellular signaling platform, which contains a cytoplasmic pattern recognition receptor, especially a nucleotide-binding oligomerization domain-like receptor (NLR). Although several types of inflammasomes have been identified so far, nucleotide-binding oligomerization domain-like receptor protein 3 (NLRP3) inflammasome is the most characteristic one 1. The researches indicated that the abnormal activation of NLRP3 inflammasome was related to the pathogenesis of various autoimmune, chronic inflammatory and metabolic diseases, including gout, atherosclerosis and type 2 diabetes 2-4.

Hydrogen sulfide (H_2_S) is an important signaling molecule that regulates many physiological and pathological processes. Recent studies indicated that H_2_S played anti-inflammatory and pro-inflammatory roles in many diseases through influencing NLRP3 inflammasome, but its mechanism was not fully understood. In this review, we summarized the recent studies on the anti-inflammatory or pro-inflammatory effects of H_2_S on NLRP3 inflammasome in a variety of diseases to provide ideas for the relevant basic research in the future.

## Overview of NLRP3 inflammasome

**Figure 1:**
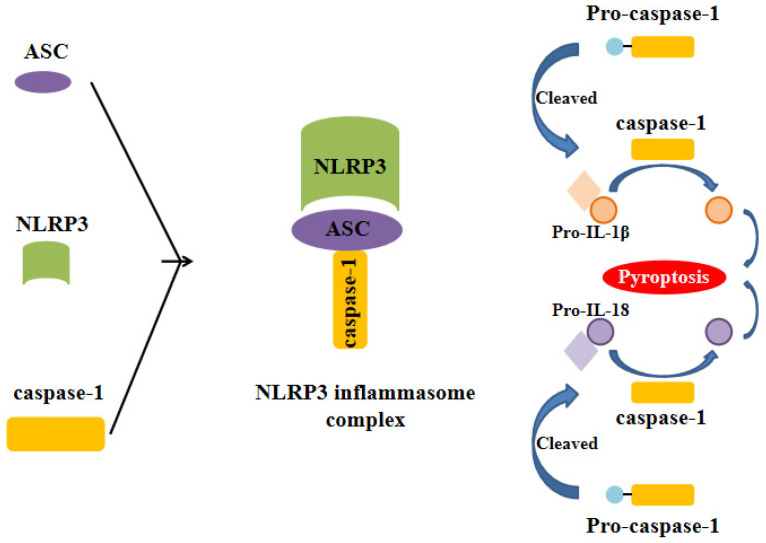
** Formation of the NLRP3 inflammasome.** The activation of NLRP3 inflammasome involves the assembling of the components of NLRP3 inflammasome (NLRP3, ASC and caspase-1) to form a complete NLRP3 inflammasome complex. This inflammasome complex allows the cleavage of pre-caspase-1 into its active isomer, caspase-1, which then cleaves pro-IL-1β and pro-IL-18 to their active isomers IL-1β and IL-18 respectively. The increase of these pro-inflammatory proteins eventually leads to pyroptosis.

Inflammasome is a complex composed of several proteins and an important part of the natural immune system. A variety of inflammasome have been found: NLRP1, NLRP2, NLRP3, NLRP6, NLRP7, NLRP12, NLRC4, IPAF and AIM2. NLRP3 inflammasome is the most thoroughly studied one, which is composed of NLRP3, apoptosis associated speck like protein (ASC) and caspase-1 precursor (pro-caspase-1) (Figure 1) 5-10. By activating caspase-1, NLRP3 inflammasome can induce the maturation and secretion of pro-inflammatory factors: Interleukin-1beta (IL-1β) and Interleukin-18 (IL-18). Mature IL-1β is an effective proinflammatory mediator in many immune responses, including the recruitment of natural immune cells to the infection site and the regulation of adaptive immune cells. Mature IL-18 plays an important role in the production of IFN-γand the enhancing the cytolytic activity of natural killer cells and T cells 11. The actived caspase-1 also induces a proinflammatory form of cell death 12. Therefore, NLRP3 inflammasome can regulate the immune response of the body and strictly control the inflammatory reaction in the pathophysiological process. NLRP3 inflammasome can be activated by different stimuli including pathogen associated molecular patterns (PAMPs) and damage associated molecular pattern (DAMPs). The first stimuli, which is mediated by proinflammatory pathways, such as toll like receptor (TLR)-mediated activation of nuclear factor-kB (NF-kB), promotes the upregulation of the components of the inflammasome. The second stimuli, including reactive oxygen species (ROS) production, intracellular potassium (K^+^) concentration and the distuption of lysosomal membrane, promotes the assembly of inflammasome and leads to the activation of caspase-1, which can catalyze the pro-IL-1β into active IL-1β 1, 9, 13-15. NLRP3 inflammasome has been reported to be related to the pathogenesis of many complex diseases, such as type 2 diabetes 16, atherosclerosis 17-20, obesity and gout 21, Alzheimer's disease and Parkinson's disease^22^, ^23^.

## Overview of H_2_S and the mechanism of H_2_S acting on NLRP3 inflammasome

**Figure 2:**
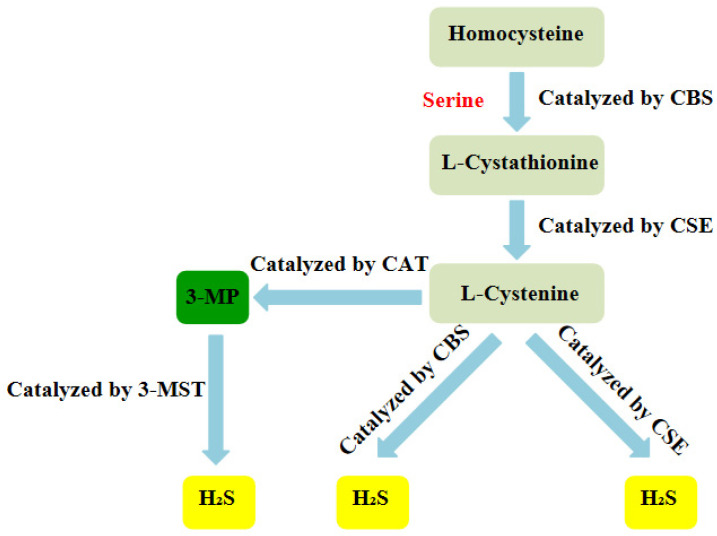
** Summary of the production process of endogenous H_2_S.** CBS:cystathionine-beta-synthase; CSE:cystathionine-gamma-lyase; 3-MST: 3-mercaptopyruvate thiotransferase; 3-MP:3-mercaptopyruvate;CAT:cysteine aminotransferase

Over the years, H_2_S has been regarded as a toxic gas with an unpleasant smell. However, since the 1990s, more and more researches have indicated that H_2_S, together with nitric oxide (NO) and carbon monoxide (CO), belongs to a class of gasotransmitters.There is increasing evidence that H_2_S can be produced in multiple organ systems in mammals, including humans and fish 24-27. In mammalian cells, H_2_S is produced by endogenous enzymatic and non-enzymatic pathways. The enzymatic generation of H_2_S, which may be important for the regulation in given cells under special conditions, is the focus of the research. Several different mammalian enzymatic systems for H_2_S production have been described in detail. Most commonly, three typical H_2_S-producing enzymes are identified: cystathionine-gamma-lyase (CSE), cystathionine-beta-synthase (CBS) and 3-mercaptopyruvate thiotransferase (3-MST). Cystathionine is produced by β-substitution reaction of homocysteine with serine catalyzed by CBS. CSE catalyzes the elimination of α, γ-cysteine of cystathionine to produce cystenine. Under the catalysis of CBS and CSE, cysteine can form H_2_S through β elimination reaction. 3-mercaptopyruvate (3-MP) is produced by transferring amines from cystine to α-ketoglutarate via cysteine aminotransferase (CAT). 3-MST catalyzes the sulphur of 3-MP to convert into H_2_S (Figure 2) 28. For the inhibition of the synthesis of endogenous H_2_S, there are several small molecular compounds, which can inhibit the synthesis of endogenous H_2_S, targeting at three kinds of H_2_S producing enzymes. Although these compounds have their limitations (potency, selectivity), these molecules, especially in combination with genetic methods, can be used to describe biological processes involving endogenous H_2_S production 29. H_2_S has the physiological functions of relaxing blood vessels, lowering blood pressure 30, 31, anti- apoptosis 32, anti-inflammation 33, anti-oxidation and regulating endoplasmic reticulum stress 34. At present, the effect of H_2_S on NLRP3 inflammasome has gradually become a research hotspot.

**Figure 3:**
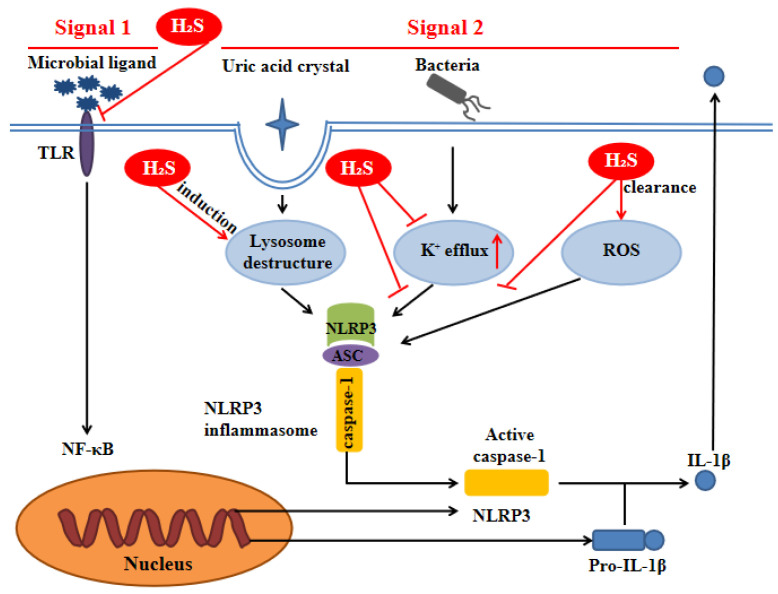
** H_2_S influences NLRP3 inflammasome through signal 1 and signal 2.** Signal 1 is mediated by microbial ligands recognized by TLR which activates the NF-kB pathway to promote the protein expression level of pro-IL-1β and NLRP3. The signal 2 promotes the assembly of the NLRP3 inflammasome complex. Under noninfectious conditions, K^+^ efflux leads to the activation of NLRP3 inflammasome. Various endogenous and exogenous particulates, including uric acid crystal, promote lysosomal damage to activate NLRP3 inflammasome. Additionally, the increase of ROS level in the cell also activates the NLRP3 inflammasome. H_2_S can influence NLRP3 inflammasome through the above pathways. ASC: apoptosis-associated speck-like protein containing a C-terminal caspase recruitment domain; NF-kB: nuclear factor kappa-light-chain-enhancer of activated B cells; ROS: reactive oxygen species; TLR:toll-like receptor

H_2_S can inhibit TLR4/NF-κB pathway 35, 36, clear ROS 37, suppress K^+^ efflux 38 and promote lysosomal membrane rupture 39, which are related to NLRP3 activation. Therefore, it can be inferred that H_2_S can act on NLRP3 inflammasome through the above pathways (Figure 3).

## H_2_S plays liver protection roles by influencing NLRP3 inflammasome

**Figure 4:**
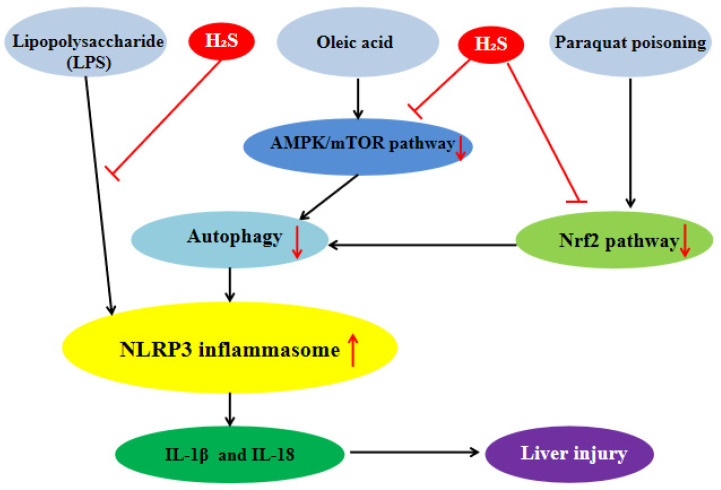
** H_2_S plays liver protection roles by influencing NLRP3 inflammasome.** H_2_S significantly inhibits NLRP3 inflammasome-mediated inflammatory injury induced by lipopolysaccharide and suppress NLRP3 inflammasome-mediated inflammatory injury induced by oleric acid through promoting autophagy via AMPK/mTOR pathway. H_2_S can alleviate NLRP3 inflammasome-mediated inflammatory injury induced by paraquat poisoning through Nrf2 signal pathway. AMPK: AMP-activated protein kinase; mTOR:mammalian target of rapamycin; Nrf2:nuclear factor erythroid-2-related factor 2

Exogenous H_2_S can inhibit the inflammatory response of hepatocytes by influencing NLRP3 inflammasome to protect liver. Our previous studies showed that in the lipopolysaccharide (LPS)-induced hepatocyte inflammation model, the protein expression level of NLRP3 inflammasome and the level of IL-1β were significantly increased and H_2_S reversed these changes, which indicated that H_2_S could significantly inhibit NLRP3 inflammasome-mediated inflammatory response 40. We also found that in the oleic acid (OA)-induced hepatocyte inflammation model, the level of autophagy was decreased significantly and the protein expression level of NLRP3 inflammasome was increased, while exogenous H_2_S could counteract the OA-induced change. 3-MA, an autophagy inhibitor, could reverse the inhibitory effect of H_2_S on NLRP3 inflammasome induced by OA, indicating that exogenous H_2_S could inhibit the protein expression of NLRP3 inflammasome by promoting autophagy in OA-induced hepatocyte. Our in-depth mechanism research showed that in OA-induced hepatocyte, H_2_S could inhibit the NLRP3 inflammasome-mediated inflammation and activate the AMP-activated protein kinase (AMPK)/mammalian target of rapamycin (mTOR) pathway and autophagy. 3-MA, an autophagy inhibitor, could counteract the effect of H_2_S, suggesting that autophagy mediated the effect of H_2_S on NLRP3 inflammasome-mediated inflammation. In addition, compound C, an AMPK inhibitor, could inhibit autophagy and counteract the anti-inflammatory effect of exogenous H_2_S. In summary, exogenous H_2_S inhibited NLRP3 inflammasome-mediated inflammation of hepatocytes through promoting autophagy via AMPK/mTOR pathway (Figure 4) 41, 42. Through consulting a large number of related literatures, we found that exogenous H_2_S could inhibit endoplasmic reticulum stress (ERS) in many diseases 28, and there was interaction between ERS and NLRP3 inflammasome 43, so whether exogenous H_2_S can inhibit NLRP3 inflammasome-mediated inflammatory response through ERS needs further study. One of the liver injuries in nonalcoholic fatty liver disease (NAFLD) is inflammatory liver injury 44, in view of our previous studies, it can be inferred that exogenous H_2_S can atteuate NAFLD by inhibiting NLRP3 inflammasome, which is still further proven. NLRP3 inflammasome will be an important target of NAFLD treatment.

Paraquat (PQ) poisoning is a serious clinical problem due to the lack of specific antidotes and the accidental or suicide PQ intake leading to high mortality. Studies have shown that oxidative stress and ROS-mediated inflammation were the main causes of PQ poisoning 45. Liver is the main source of endogenous antioxidants and plays an important role in enzyme metabolism and detoxification. Therefore, the liver is more vulnerable to PQ poisoning 46, 47. It has been reported that PQ activated NLRP3 inflammasome, resulting in the secretion of IL-1β and IL-18 in macrophages. Therefore, the inhibition of NLRP3 inflammasome-mediated inflammatory response may be beneficial to the treatment of PQ poisoning 48, 49. Zhenning Liu et al.found that in PQ-induced rat liver injury, H_2_S could significantly inhibit the protein expression level of NLRP3 inflammasome, pro-caspase-1 and the secretion of IL-1β and activate Nrf2 signal pathway. The nuclear factor erythroid-2-related factor 2 (Nrf2) gene knockout or siRNA-Nrf2 could counteract the protective effect of H_2_S, suggesting that H_2_S could alleviate PQ-induced liver injury by inhibiting NLRP3 inflammasome-mediated inflammatory response through Nrf2 signal pathway (Figure 4)^50^. The inhibitory effect of H_2_S on NLRP3 inflammasome has therapeutic effect on PQ-induced liver injury.

## H_2_S plays renal protection role by influencing NLRP3 inflammasome

Acute renal injury is a clinical syndrome caused by many factors, which is characterized by rapid decline of renal function 51. It has been reported that NLRP3 inflammasome participated in the inflammatory process, which might be the key to the development of acute renal injury 52. Yuhong Chen, et al. found that exogenous H_2_S could inhibit the protein expression level of NLRP3 inflammasome to attenuate LPS-induced rat acute renal injury 53. The signal transduction mechanism of the above-mentioned action of H_2_S needed further study. Renal fibrosis and renal injury are important clinical features of many chronic kidney diseases (CKDS) 54. It has been shown that NLRP3 inflammasome was involved in the pathogenesis of CKDS 55, 56. In the model of injury and fibrosis of unilateral ureteral obstruction (UUO) mice, exogenous H_2_S could alleviate macrophage infiltration, tissue fibrosis, and inhibit NF-κB and IL-4/signal transducer and activator of transcription 6 (STAT6) signaling pathways and NLRP3 inflammasome, and NLRP3 inhibitor had the effect similar to that of H_2_S, which suggested that H_2_S alleviated renal fibrosis via inhibiting NLRP3 inflammasome. These studies also showed that NLRP3 inflammasome activation contributes to macrophage infiltration and tissue fibrosis, and NF-κB and IL-4/STAT6 signaling pathways were related to macrophage infiltration. So, it could be inferred that H_2_S alleviates renal fibrosis in response to UUO by suppressing macrophage infiltration through inhibition of NLRP3 inflammasome via NF-κB and IL-4/STAT6 signaling pathways, which needed to be further proven 57-59.

## H_2_S plays neuroprotection role by influencing NLRP3 inflammasome

Intracerebral haemorrhage (ICH) is a devastating stroke with high mortality and incidence rate. Countless evidences from preclinical and clinical studies suggested that inflammatory mechanisms were involved in ICH-induced secondary brain injury 60, 61. Studies have shown that the activation of NLRP3 inflammasome played an important role in the development of neuroinflammation after ICH 62. Exogenous H_2_S could inhibit the activation of NLRP3 inflammasome and the subsequent release of IL-1β induced by ICH. Purinergic P2X7 receptor (P2X7R) is an ATP gated, non-selective cation channel, belonging to the family of ionotropic P2X receptors. It was reported that P2X7R interacts with NLRP3 inflammasome, which was responsible for the recruitment and activation of NLRP3. H_2_S could suppress the P2X7R expression and the overexpression of P2X7R could upregulate the expression of NLRP3 inflammasome on microglia after ICH. These results suggested that H_2_S could suppress NLRP3 inflammasome-mediated neuroinflammation by inhibiting P2X7 receptor after ICH in rats 63. As we all know, H_2_S plays an important role in antioxidation, therefore, it is reasonable to speculate that in addition to inhibiting the expression of P2X7R, H_2_S can also inhibit NLRP3 inflammasome-mediated neuroinflammation by directly eliminating the ROS after ICH, which still needs to be studied. Ischemic stroke is one of the main causes of the disability and death worldwide 64. Inflammatory response was often involved in ischemic stroke injury 65. H_2_S could play a neuroprotective role by inhibiting the activation of NLRP3 inflammasome in ischemic brain 66.The inhibitory effect of H_2_S on NLRP3 inflammasome has potential therapeutic value for ischemic stroke injury.

## H_2_S inhibits NLRP3 inflammasome in macrophages

Fatty acids (FA) have been shown to induce inflammation in primary human macrophages 67. In FA-induced RAW264.7 cell, the protein expression level of NLRP3 inflammasome and the level of IL-1β and IL-18 were increased and the the TLR4/ NF-κB pathway was activated, while H_2_S could counteract these changes. NLRP3 siRNA reduced the level of IL-1β and IL-18 induced by FA, suggesting that NLRP3 inflammasome mediated FA-induced inflammation. TLR4 inhibitor and NF-κB inhibitor reduced the protein expression level of NLRP3 inflammasome induced by FA, suggesting that TLR4/NF-κB mediated the activation of NLRP3 inflammasome induced by FA. In summary, it could be inferred that exogenous H_2_S suppressed NLRP3 inflammasome-mediated inflammation by inhibiting TLR4/NF-κB pathway in FA-induced RAW264.7 cells, which needed further study 35. It has been shown that mitochondrial uncoupling protein 2 (UCP2) regulated NLRP3 inflammasome by inducing lipid synthesis in macrophages 68, So whether exogenous H_2_S could regulate lipid synthesis pathway by inhibiting NLRP3 inflammasome needed further research. The activation of NLRP3 inflammasome in macrophages has been considered to be involved in diseases 69-75. Exogenous H_2_S could inhibit NLRP3 inflammasome-mediated inflammation in human macrophages exposed to H_2_O_2_, which was related to the reduction of mitochondrion ROS (mtROS). The in depth study on the mechanism of the above effect showed that H_2_S decreased the production of mtROS by S-sulfhydrating c-Jun at cysteine-269. The suppression of S-sulfhydrated c-Jun of H_2_S could reverse the inhibition of H_2_S on NLRP3 inflammasome, suggesting that H_2_S inhibited the NLRP3 inflflammasome activation via sulfhydration of c-Jun at cysteine-269. The S-sulfhydrated c-Jun of H_2_S increased SIRT3 expression, and in the macrophages of SIRT3-/- mice exposed to H_2_O_2_, the inhibition of H_2_S on NLRP3 inflammasome was diminished, which suggested that H_2_S inhibited NLRP3 inflammasome through SIRT3 76. The modification of c-jun by H_2_S may provide ideas for the treatment of NLRP3 inflammasome involved diseases. In primary human macrophages, H_2_S inhibited monosodium urate (MSU)-induced NLRP3 inflammasome activation, xanthine oxidase (XO) activity and mtROS generation while febuxostat (a XO-inhibitor) diminished MSU-induced mtROS generation and NLRP3 inflammasome activation, which suggested H_2_S was capable of inhibiting NLRP3 inflammasome by suppressing XO activity 77, 78.

## H_2_S plays a protective role by influencing NLRP3 inflammasome in diabetes

Chronic, low-level systemic and aseptic inflammation is a common feature of diabetic cardiomyopathy (DCM) 79. A study showed that inhibiting NLRP3 inflammasome could significantly alleviate DCM 80. H_2_S has been reported to protect cardiomyocytes from inflammation and cell death in diabetic models 81, 82. In high glucose(HG)-induced H9c2 cardiac cells, the protein expression level of NLRP3 inflammasome and the level of IL-1β and IL-18 were increased and the the TLR4/NF-κB pathway was activated, while H_2_S could counteract these changes. NLRP3 siRNA reduced the level of HG-induced IL-1βand IL-18, indicating that NLRP3 inflammasome mediated HG-induced inflammation. TLR4 inhibitor and NF-κB inhibitor reduced the protein expression level of HG-induced NLRP3 inflammasome, indicating that TLR4/NF-κB mediated the activation of NLRP3 inflammasome in HG-induced H9c2 cardiac cells. In summary, it could be inferred that exogenous H_2_S suppressed NLRP3 inflammasome-mediated inflammation by inhibiting TLR4/NF-κB pathway in H9c2 cardiac cells, which needed further study 36. HG could cause lipid metabolism disorder, and NLRP3 inflammasome participated in lipid metabolism process 70, so whether H_2_S could improve lipid metabolism through NLRP3 inflammasome to alleviate DCM needed further study. Diabetic retinopathy is a common complication of diabetes mellitus, which is also the main cause of visual impairment and blindness 83. Chronic hyperglycemia damaged not only the retinal vessels but also the retinal pigment epithelial cells (RPE)^84^. In HG-induced RPE cells, HG increased the production of intracellular ROS and the level of IL-1β and IL-18 and activated NLRP3 inflammasome while H_2_S counteracted these changes. Knock down of NLRP3 decreased the level of IL-1β and IL-18, suggesting that NLRP3 inflammasome mediated the HG-induced inflammation. In conclusion, H_2_S inhibits HG-induced inflammation of human retinal pigment epithelial cells through inhibiting NLRP3 inflammasome 85. In HG-induced 3T3‑L1 adipocytes, H_2_S has the effect similar to the above 86. H_2_S has therapeutic effect on diabetes through inhibiting NLRP3 inflammasome. Diabetes-accelerated atherosclerosis is the most common cardiovascular complication of diabetes mellitus 87. H_2_S also decreased the HG-induced endothelial injury and the protein expression level of NLRP3 inflammasome in vivo and in vitro, while the silencing of NLRP3 had the effect similar to that of H_2_S, suggesting that H_2_S protected against diabetes-accelerated atherosclerosis by inhibiting the activation of NLRP3 inflammasome 88. It provided the new evidences for the treatment of cardiovascular diseases with H_2_S. NLRP3 inflammasome is related to lipid metabolism, and H_2_S can promote lipolysis 89, so It can be deduced that H_2_S can promote lipolysis by inhibiting NLRP3 inflammasome against diabetes-accelerated atherosclerosis, which needs further study.

## H_2_S plays a protective role in other inflammatory reactions by inhibiting NLRP3 inflammasome

Repeated exposure of mice to high concentrations of ozone has been shown to cause chronic lung inflammation, emphysema and airflow restriction 90. In ozone exposed mice, ozone increased the protein expression level of the NLRP3 inflammasome, cleavage caspase-1 and the level of p38 mitogen-activated protein kinases (MAPK) phosphorylation and decreased the level of protein kinase B (Akt) phosphorylation, while H_2_S counteracted these changes 91. Therefore, it can be inferred that H_2_S can alleviate lung inflammation caused by ozone exposure through supressing NLRP3 inflammasome and p38MAPK/Akt pathways, which needs to be proven by using specific inhibitors or specific knock-out mice to block certain pathways. It is reported that NLRP3 inflammasome mediated dextran sodium sulfate (DSS)-induced colitis. H_2_S could reduce the inflammation of colitis induced by DSS through inhibiting the activation of NF- κB pathway, so it could be infered that H_2_S could relieve DDS-induced colitis through suppressing NLRP3 inflammasome via NF- κB pathway, which neede further study 92-95. In DSS-induced colitis, H_2_S decreased the protein expression level of NLRP3 inflammasome, pro-caspase-1, and Nrf2 and the silencing Nrf2 has the effects similar to the above, which indicated that H_2_S inhibited NLRP3 inflammasome through Nrf2 pathway 96. H_2_S could ameliorate endothelial dysfunction and hypertension 97, 98. The mechanism research showed that H_2_S could improve endothelium-dependent contraction and relaxation and reduce the protein expression levels of NLRP3 inflammasome and the level of IL-1β in spontaneously hypertensive rats. The above ameliorative effects of H_2_S were abolished by LPS (a NLRP3 activator), suggesting that H_2_S ameliorated endothelial dysfunction and hypertension by inhibiting NLRP3 inflammasome 99. It suggested that the effect of H_2_S on NLRP3 has potential therapeutic function in the treatment of hypertension.

## H_2_S promotes NLRP3 inflammasome to promote diseases development

Besides the anti-inflammatory effect, H_2_S can also promote the inflammatory reaction to participate in the development of diseases through promoting NLRP3 inflammasome. The studies showed that in human monocyte, H_2_S could induce NLRP3 inflammasome dependent secretion of IL-1β and IL-18 by promoting the assembly of NLRP3 inflammasome to contribute to diseases development 100, 101. In broiler thymus, the atmospheric H_2_S could activate NLRP3 inflammasome to decrease thymus index, thymus immunoglobulin and T lymphocyte number and damaged thymus morphology, which suggested that the atmospheric H_2_S has immunotoxicity. The mechanism study of the above actions showed that TLR-7/myeloid differentiation factor 88(MyD88)/NF- κB pathway was activated by H_2_S. So It can be inferred that H_2_S might activate NLRP3 inflammasome via TLR-7/MyD88/NF-κB pathway, which needed to be further proven 102.Under what conditions does H_2_S promote NLRP3 needs further study.

## Summary

H_2_S has both anti-inflammatory and pro-inflammatory effects and the mechanism has not been fully studied. The current researches have showed that the mechanism of H_2_S in inflammation was related to the concentration of H_2_S, the stage of development of inflammatory diseases and the types of tissues affected by H_2_S. For example, the low concentration of H_2_S can inhibit the inflammatory response to reduce the inflammatory damage of tissues and organs, while the high concentration of H_2_S can promote the inflammatory response to aggravate the inflammatory damage. Similar to the above, the effects of H_2_S on NLRP3 inflammasome are either inhibition or promotion. Whether H_2_S can inhibit NLRP3 inflammasome to play a protective role or promote NLRP3 inflammasome to participate in the development of diseases, especially the latter, needs further study. No matter what role H_2_S plays, the research and development of H_2_S donor or H_2_S inhibitor related drugs will provide a new way for the treatment of inflammatory diseases. In addition, the mechanism of H_2_S acting on NLRP3inflammasome has not been fully studied. For example, whether H_2_S can act on NLRP3 inflammasome by influencing lysosomal rupture or K^+^ efflux remains to be elucidated.

In conclusion, NLRP3 inflammasome may be a potential target for H_2_S therapy in inflammatory diseases with the in-depth study of the effect of H_2_S on NLRP3 inflammasome.
